# Comparison of Drying Techniques to Produce Stable and Bioavailable Encapsulated ACE-2 Nanoparticles

**DOI:** 10.3390/pharmaceutics17040537

**Published:** 2025-04-21

**Authors:** Yigong Guo, Alberto Baldelli, Dai Shi, David D. Kitts, Anubhav Pratap-Singh, Anika Singh

**Affiliations:** 1 Natural Health and Food Products Research Group, Centre for Applied Research and Innovation (CARI), British Columbia Institute of Technology, 4355 Mathissi Pl, Burnaby, BC V5G 4S8, Canada; yigong_guo@bcit.ca; 2 Food, Nutrition, and Health, Faculty of Land & Food Systems, 2205 East Mall, University of British Columbia, Vancouver, BC V6T 1Z4, Canada; a.baldelli@uq.edu.au (A.B.); das752@student.ubc.ca (D.S.); david.kitts@ubc.ca (D.D.K.); anubhav.singh@ubc.ca (A.P.-S.)

**Keywords:** ACE-2, Nanoparticles, Spray Freeze Dry (SFD), Freeze Dry (FD), Spray Dry (SD)

## Abstract

**Background/Objectives:** COVID-19 infection continues globally, with frequent emergence of unfamiliar SARS-CoV-2 variants acting to impair immunity. The competitive binding of SARS-CoV-2 spike proteins and angiotensin-converting enzyme 2 (ACE-2) can decrease the binding of the virus on native ACE-2 receptors on healthy human cells. It remains a practical approach to lessen viral spread. In this study, a method to encapsulate ACE-2 in the form of chitosan/tripolyphosphate cross-linked nanoparticles (NPs) was developed with emphasis placed on the best dehydration method to secure functional ACE-2 nanoparticles. **Methods:** Methods: Preparation conditions were assessed by varying pH (4.0–6.5) and the ratio between chitosan and ACE-2 mixing ratios (1:1, 1.5:1, 2:1, 2.5:1, and 3:1). The formulated NPs were then dehydrated using different approaches that included spray-drying (SD), freeze-drying (FD), and spray-freeze drying (SFD) and used varying mannitol concentrations (0, 1:1, and 5:1 of total weight). The mannitol was served as a cryoprotectant in this study. **Results:** The best formulation achieved used a pH 5.5 with a mixing chitosan–ACE-2 ratio of 2:1, where ACE-2-loaded NPs had an average particle size of 303.7 nm, polydispersity index (PDI) of 0.21, encapsulation efficiency (EE) of 98.4%, and ACE-2 loading content (LC) of 28.4%. After reconstitution, all SD samples had a relatively low yield rate, but the ACE-2 NPs dehydrated specifically using SFD required a lower amount of added mannitol (1:1 of its total weight) and produced a higher yield rate (*p* < 0.05) and similar PDI and EE values, along with relatively good particle size and LC. This formulation also produced a high ACE-2 release and uptake in differentiated Caco-2 cells, thus representing an effective ACE-2 encapsulation procedure for use with dry powders. **Conclusions:** This work showed that spray-freeze drying was the best method to dehydrate ACE-2 NPs, using less cryoprotectant to create a significant advantage in terms of greater loading capacity with lower additive requirements.

## 1. Introduction

Although COVID-19 cases are now lower than during the pandemic stage, this contagious disease continues to affect global populations with varying transmission rates and severity across regions. The ongoing emergence of SARS-CoV-2 variants renders existing vaccines less effective, underlining the pressing need for new therapeutic strategies to control this disease [1]. SARS-CoV-2 invades the human body through the binding interaction between the surface spike proteins and the angiotensin-converting enzyme 2 (ACE-2) receptors present on healthy human cells, involved with triggering virus–cell fusion and the release of viral genetic materials for replication [2,3]. Studies have shown that ACE-2 is a functional receptor for SARS-CoV-2 and HCoV-NL63, which initiate endocytosis-driven cell entry. Likewise, ACE-2 can be used in the host cell entry mechanisms of SARS-CoV-2 [4]. As mutated SARS-CoV-2 tends to retain a high binding affinity to the ACE-2 receptors, therapeutic approaches that block or reduce the occurrence of such interactions remain practical approaches to lessen COVID-19 infection [1,5]. Although effective in reducing viral spread, the primary intravenous administration route of ACE-2 derivatives requires frequent supplementation to maintain a sufficient drug concentration due to a rapid degradation in free forms [1,6]. Intravascular delivery also exhibits low transportation rates across plasma-lung barriers and is considered an inconvenient delivery route, further limiting therapeutic efficacy [1].

To find a solution to enhance the effectiveness of exogenous ACE-2, nanocarrier systems have been explored for their protective role in aiding the absorption-enhancing capacity of embedded drugs. It can be inferred that the majority of ACE-2 nanocarrier studies indeed target application to respiratory epithelial cells—logically so, given that COVID-19 is a primarily respiratory illness. However, since the levels of ACE-2 receptors distributed in the gastrointestinal (GI) tract far exceed those in the respiratory tract, the oral route of infection could be a strategy to establish a viable approach with designed novel ACE-2-based therapeutic approaches. Moreover, to minimize SARS-CoV-2 spread within the human body more comprehensively, GI delivery of exogenous ACE-2 warrants a more in-depth investigation.

Chitosan, being a naturally occurring cationic polysaccharide (pKa of ~6.5), is highly mucoadhesive to negatively charged surfaces such as those in the GI tract, thus prolonging the contact with target tissues and enhancing drug delivery when presented as an encapsulation matrix [7,8]. These electrostatic interactions also enhance the permeability of biological membranes by opening the epithelial tight junctions to improve the absorption of poorly permeable compounds via both the paracellular and transcellular transportation routes [8]. The preparation of chitosan-based NPs typically includes emulsification, precipitation, and ionic gelation methods, the last of which is advantageous because it is a relatively simple and mild procedure that does not require application of organic solvents or high temperatures [9]. When suspended in aqueous solutions, cationic chitosan readily complexes with anionic agents, a popular one of which is tripolyphosphate (TPP). Nanoparticles generated from chitosan/TPP mixtures generally have uniform particle size, low cellular toxicity, and high encapsulation efficiency and loading capacity [10]. This technique has been extensively used to encapsulate natural bioactives, synthetic drugs, and therapeutic proteins and peptides [10]. To date, there has been no report on the successful encapsulation of ACE-2 using chitosan/TPP NPs.

Ingredient dehydration (e.g., powder production) is a common practice in the pharmaceutical and food industries to preserve the desirable characteristics of products while also extending shelf-life, thus saving on packaging, transportation, and storage costs. Likewise, freshly prepared NPs are typically dehydrated into dry powder forms before they are ready for use directly or added into other formulations. Spray-drying (SD) is the most applied dehydration technique for this purpose, as it is an economic, quick, and continuous process [11,12]. The technique involves the atomization (spraying) of a feed solution into a drying chamber where hot air is circulated. Due to the high temperatures required to immediately evaporate water, thermal damage can occur, especially with heat-sensitive compounds (e.g., proteins). A popular alternative to avoid ingredient thermal degradation is to use freeze-drying (FD), where mass (water) transfer is executed by the sublimation of ice crystals within the frozen feed stock. However, due to the long processing time (2.5 times higher than SD) and the high energy input required to deliver lower-pressure environments, the process is relatively expensive [11,13]. To overcome the limitations that come with both SD and FD, a combination approach termed ‘spray-freeze drying’ (SFD) has been developed. Similar to SD, the feed solution is first atomized into fine droplets directly into a cryogenic zone filled with liquid nitrogen, where the frozen droplets are collected for subsequent drying following the typical FD protocol. By reducing product dimensions through spraying, mass transfer in the freeze dryer is significantly enhanced; hence, an overall shorter process with lower costs is realized [13]. The quick freezing involved with SFD also allows particles to retain a spherical shape while achieving a large, highly porous specific surface area; the net result is a drastically improved reconstitution ability [14] and drug release [15].

The overarching goal of the present research was to produce high-quality dry encapsulated ACE-2 contained within a chitosan/TPP NP matrix. Formulation variables were tested by varying pH (4.0–6.5) and chitosan/ACE-2 mixing ratios (1:1, 1.5:1, 2:1, 2.5:1, and 3:1) to achieve suitable particle size, drug loading, and entrapment efficiency. To achieve the best dehydration conditions of prepared ACE-2 NPs, three different drying methods, namely spray-drying (SD), freeze-drying (FD), and spray-freeze drying (SFD), with different mannitol concentration ratios (e.g., 0, 1:1, and 5:1) were explored. The outcomes assessed included morphological properties, yield rate, reconstitution (rehydration), stability, in vitro release, cellular uptake, and toxicity assessment in cultured Caco-2 cells.

## 2. Materials and Methods

### 2.1. Materials

The ACE-2 used in this study was kindly provided by Dr. David Perrin, UBC Chemistry (Vancouver, BC, Canada). Low MW chitosan (average MW 100 KDa, 75–85% deacetylated), TPP, and essential cell culture medium were purchased from Sigma-Aldrich (Oakville, ON, Canada). All other reagents used were of analytical grade or HPLC grade. Caco-2 cells (HTB-37, American Type Culture Collection, Manassas, VA, USA) were cultured in Dulbecco’s Modified Eagle Medium (DMEM) media (Invitrogen, Carlsbad, ON, Canada) and supplemented with 10% fetal bovine serum (FBS) and 100 U/mL of penicillin and streptomycin, respectively (Sigma, St. Louis, MO, USA).

### 2.2. ACE-2 Nanoparticle Preparation

The ACE-2 nanoparticles (NPs) were prepared using an ion-gelation method. Briefly, ACE-2 was initially mixed with a TPP solution, after which a chitosan solution was added dropwise under high-speed stirring (10,000 rpm) using a Polytron PCU-2-110 high-speed homogenizer (Brinkmann Ind., Westbury, NY, USA). After pH adjustment, the mixture was maintained under high-speed stirring (15,000 rpm) in an ice bath for 30 min to achieve crosslinking of the ACE-2 NPs. Subsequently, the samples underwent sonication for an additional 30 min in an ice bath to protect them from breaking using a probe-type ultrasonicator (UP 200ST, Hielscher Ultrasonics, Teltow, Germany) to reduce the particle size. The ACE-2 NPs were then prepared at six different pH values (4–6.5) with varying ratios (1:1, 1.5:1, 2:1, 2.5:1, and 3:1) between chitosan and ACE-2 to optimize the nanoparticle formulation.

### 2.3. ACE-2 NP Dehydration

Three dehydration techniques were used to prepare ACE-2 NP powder: freeze-drying (FD), spray-drying (SD), and spray-freeze drying (SFD). Mannitol was added to each sample at ratios of 0, 1:1, and 5:1 of total NP weight. A Labconco FreeZone freeze-dryer equipped with tray dryers (Labconco, Kansas City, MO, USA) was used to prepare FD ACE-2 NPs. The temperature and vacuum pressure were set at −10 °C and 0.350 Torr, respectively, for the first 2 h, and at 0 °C and 0.120 Torr, respectively, for the remaining 22 h to obtain dry ACE-2 NPs. For SD, a Buchi mini spray dryer B-290 (BÜCHI, Flawil, Switzerland) was used. The drying parameters selected were inlet temperature of 90 °C, outlet temperature of 40 °C, a feeding flow rate of 6 mL/min, and an airflow rate of 4 L/min. For SFD, the drying chamber of the spray dryer was replaced with a laboratory jacket containing a cylindrical borosilicate glass Dewar flask (250 mL, StonyLab, Nesconset, NY, USA) filled with liquid nitrogen. The distance between the atomizer and the surface of the liquid nitrogen was set at 10 cm. The ACE-2 NPs were directly sprayed into the liquid nitrogen at a feeding flow rate of 5 mL/min and an airflow rate of 4 L/min. Frozen ACE-2 NPs were further dehydrated using a freeze dryer, and the same set of parameters mentioned above were used. In this paper, “−”, “+”, and “++” were used to indicate the amount of mannitol added into the NPs, where “−” = no mannitol; “+” = low mannitol; and “++” = high mannitol.

### 2.4. Characterization of Nanoparticles

The mean diameter, polydispersity index (PDI), and zeta potential of ACE-2 NPs were determined by dynamic light scattering (DLS) using a Litesizer 500 (Anton Paar, Graz, Austria). All samples were suspended in DD water for the tests of mean diameter, polydispersity index (PDI), and zeta potential. A scanning electron microscope (SEM) (Hitachi S4700 SEM, field emission gun ultra-high resolution SEM) was used to measure the morphology of dehydrated ACE-2 NPs. The settings of 10 Kv and 8 mA were applied to all samples. To improve the quality of the figures, samples were coated with an 8 nm coating of gold using a Cressington Sputter Coater. To evaluate the encapsulation efficiency (EE) of ACE-2 NPs, ultrafiltration tubes with a MW cutoff of 100 kDa were used, and ACE-2 NPs were added and centrifuged at 500× *g* for 30 min. The free ACE-2 present in the filtrate was quantified using an Agilent 1100 series HPLC system (Agilent, Santa Clara, CA, USA) with a quaternary pump, an autosampler, a column heater, and a diode array detector. ACE-2 was quantified from a C18 column (Zorbax, 3.5 μm, 4.6 mm × 150 mm, Agilent, Santa Clara, CA, USA) and detected at 220 nm. The mobile phase consisted of acetonitrile and water with 0.1% trifluoroacetic acid, running at a gradient ratio of 10:90 to 100:0 for 10 min. The area under the curve (AUC) was used to determine the concentration of the ACE-2 in the samples. The EE of the ACE-2 NPs in percentages were calculated using Equation (1). The loading content (LC) of the ACE-2 NPs was calculated as the ratio between the total weight of loaded ACE-2 and the total weight of ACE-2 NPs, as shown in Equation (2).

(1) EE%=1−free ACE−2Total ACE−2×100%

(2) LC%=Weight of loaded ACE−2Weight of NPs×100%

### 2.5. Fourier Transform Infrared–Attenuated Total Reflectance (FTIR-ATR) Spectroscopy

Free ACE-2, chitosan, freshly prepared ACE-2 NPs, and ACE-2 NPs dehydrated using different methods were analyzed using FTIR–ATR spectroscopy. Spectral characterization was performed with a Spectrum 100 FTIR spectrophotometer (PerkinElmer, Waltham, MA, USA) equipped with universal ATR sampling accessories. The spectra were recorded by averaging 16 scans over a frequency range of 4000–600 cm^−1^ with a resolution of 4 cm^−1^.

### 2.6. Reconstitution Test

All dehydrated ACE-2 NPs were reconstituted in double-distilled water for a reconstitution test. The particle size, polydispersity index (PDI), encapsulation efficiency (EE), and loading capacity (LC) were evaluated using the same methods as previously described to assess the quality after reconstitution. Additionally, the yield of ACE-2 was calculated by comparing recovery after reconstitution.

### 2.7. In Vitro Release Profile of ACE-2 Loaded NPs

The in vitro release behavior of freshly prepared and dehydrated ACE-2 NPs was tested using a dialysis bag method (MW cutoff of 100 kDa, Spectra Por Inc., Rancho Dominguez, CA, USA) [16]. Freshly prepared and redissolved dried ACE-2 NPs were dialyzed into pH 2.5, 6.0, and 7.0 (0.1 M phosphate-buffered saline, PBS) model solutions with pepsin, thereby representing the pH environment of the stomach, duodenum, and upper small intestine, respectively. The samples were dispersed in a dialysis bag containing 10 mL model solutions. All samples were incubated at 37 °C with continuous shaking at 200 rpm, and 5 mL of the liquid outside the dialysis bag was withdrawn at the following time points: 0.5, 1, 2, 3, 4, and 6 h. The volume was immediately replenished with fresh dialysis model solution. The content of the ACE-2 was measured by HPLC, and the rate of ACE-2 release from the NPs was calculated as the ratio between released free ACE-2 and the total amount of ACE-2 encapsulated in the NPs.

### 2.8. In Vitro Cellular Uptake Study

The in vitro cellular uptake study was performed according to our previous study [12]. Briefly, free ACE-2 and ACE-2 NPs dehydrated by different methods were tested for cellar uptake using a cell lysis method that included Caco-2 cells seeded at a density of 5 × 10^4^ cells/cm^2^ in a 24-well plate and cultivated for 21 days to establish differentiation before testing. Cells were treated with free ACE-2 and ACE-2 NPs dehydrated by different methods, respectively, for 6 h, and then extracted by lysis buffer (50 mM Tris, pH 7.4, 250 mM NaCl, 5 mM EDTA, 50 mM NaF, 1 mM Na_3_VO_4_, 1% NP-40, and 0.02% NaN_3_) to release intracellular ACE-2 for subsequent analysis using HPLC with pure ACE-2 standard.

### 2.9. In Vitro Cytotoxicity Assay

The MTT test was used to assess the cytotoxicity of different concentrations of dehydrated ACE-2 NPs. Differentiated Caco-2 cells were seeded at a density of 5 × 10^4^ cells/cm^2^ in 96-well plates. Free ACE-2 and ACE-2 NPs dehydrated by different methods were diluted in concentrations ranging from 50 to 500 μg/mL in culture medium and then applied to the cells. After a 24 h incubation, the cells were washed and refreshed with a medium containing 0.5 mg/mL of MTT, followed by another 2 h of incubation. Cytotoxicity was measured by recording the enzymatic reduction of yellow tetrazolium MTT to purple formazan at 550 nm using a Tecan Infinite M200 Pro spectrophotometer plate reader (Tecan, Mennedorf, Switzerland).

### 2.10. Statistical Analysis

All experiments were performed in triplicate (*n* = 3). The values are presented as mean  ±  standard deviation. All data were tested for normal distribution prior to conducting a one-way ANOVA, or a paired *t*-test using IBM SPSS Statistics 26 (IBM, Endicott, NY, USA).

## 3. Results and Discussion

### 3.1. Optimization of Chitosan–TPP Cross-Linked ACE-2 NPs

**Figure 1:**
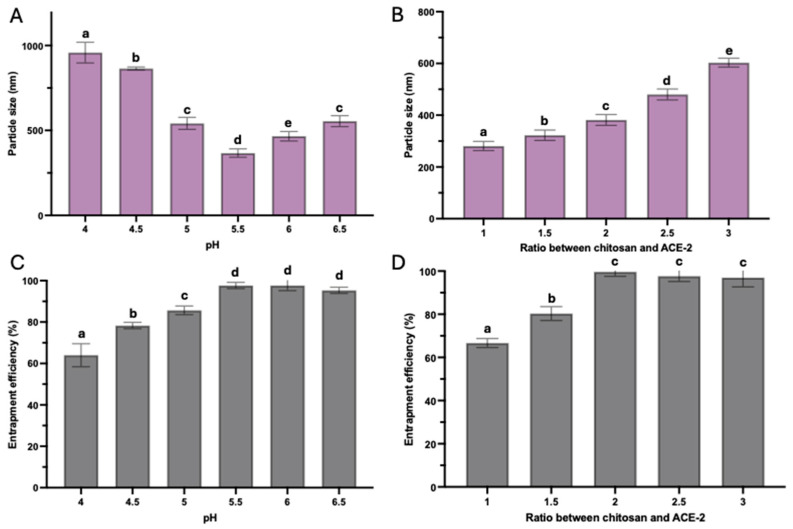
Effect of pH on particle size (prepared at the 3:1 mass ratio of chitosan and ACE-2) (**A**); effect of mass ratio between chitosan and ACE-2 on particle size (prepared at pH 6) (**B**); effect of pH on entrapment (encapsulation) efficiency (prepared at the 3:1 mass ratio of chitosan and ACE-2) (**C**); and effect of mass ratio between chitosan and ACE-2 on entrapment (encapsulation) efficiency (prepare at pH 6) (**D**). Values represent means ± SD (*n* = 3). Letters a–e denote significantly different (*p* < 0.05) groups.

It was critical to control the pH of the mixing solution and the ratio between ACE-2 and chitosan because of direct impacts on the formation of the NPs that influenced the final particle size and EE. Particle size exhibited a high association with the pH of the mixing solution (Figure 1A). The mean particle size (nm) of ACE-2-loaded NPs was reduced and the EE was increased when the pH of the system increased from 4.0 to 5.5, while the mean particle size started to increase and the EE remained the same once the pH further increased to 6.5 (Figure 1A,C). Additionally, as the chitosan-to-ACE-2 ratio increased, the mean particle size also tended to increase (Figure 1B). Chitosan is a weak polyelectrolyte with a pKa value of ~6.5. In acidic media, the presence of a predominant amino group is protonated by hydrogen ions, thus exhibiting a positive charge. Therefore, this condition is often used as a carrier to encapsulate negatively charged molecules. In this study, chitosan was used to encapsulate ACE-2 with an isoelectric point of 5.4 [17] to investigate the effect of pH on chitosan NP particle size. A continuous decrease in particle size before the pH reached approximately 5.5, and a significant increase in size was observed when pH > 5.5 (Figure 1A). This phenomenon was due to the ACE-2 molecules acquiring a negative surface charge as the pH increased, which facilitated electrostatic interactions with the chitosan/TPP complex and, thus, resulted in a decrease in particle size and an increase in EE. However, adjusting the pH to 6.5 led to the deprotonation of the amino groups on chitosan, causing folding. Thus, higher pH exposed fewer amino ions to TPP and ACE-2, resulting in less cross-linking and, ultimately, a larger particle size and lower EE. Furthermore, the higher the chitosan content, the more ACE-2 can be encapsulated. Since chitosan acted as a coating material, increasing the relative proportion would correspond to an increased thickness of the NP outer layer, resulting in a larger particle size and higher EE. In this case, the highest EE was reached when the chitosan-to-ACE-2 ratio reached 2:1, and no significant change in EE was observed, with further increase in the ratio (Figure 1D). Therefore, the optimal preparation condition obtained in this study was at pH 5.5 and an ACE-2/chitosan mass ratio of 2:1 to prepare ACE-2-loaded NPs for further studies. Under this preparation condition, ACE-2 NPs had an average particle size of 303.7 nm, PDI of 0.21, EE of 98.4%, zeta potential of 6.8 mV, and ACE-2 LC of 28.4% (Figure S1).

### 3.2. Morphological Analysis of ACE-2 NPs Dehydrated by Different Methods

**Figure 2:**
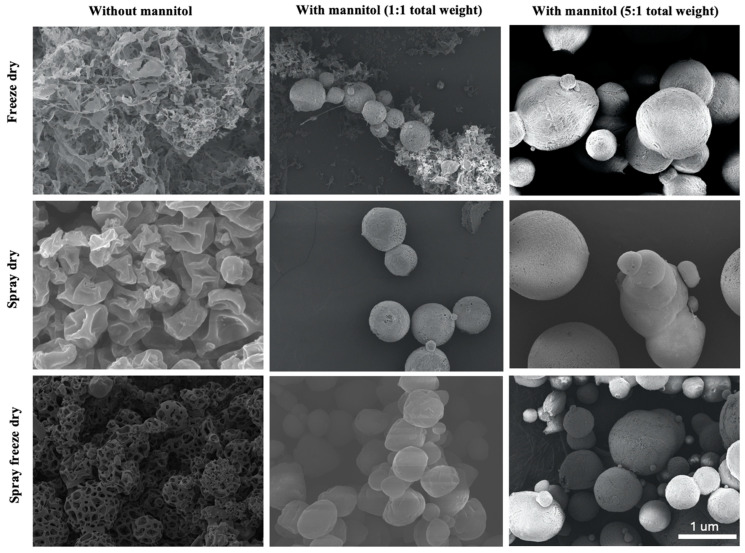
Morphology (SEM) of ACE-2 NPs dehydrated by different methods.

The analysis of the morphological characteristics of dried ACE-2NPs prepared using FD, SD, and SFD were performed to select the best dehydration technique for powder formation. The preferred approach should include drug stability, uniform particle shape, high drug loading, and good reconstitution ability. In this study, as FD techniques were involved, we also evaluated the impact of mannitol as a bulking agent. Hence, in all three dehydration techniques examined, mannitol had a dual role as a filler and as a cryoprotector to maintain the spherical morphology of the ACE-2 NPs [18]. ACE-2 NPs dried without mannitol present and with mannitol at a weight ratio of 1:1 and 5:1 was compared. For FD, ACE-2 NPs without mannitol produced a highly porous structure with an irregular and rough surface, as shown by SEM, in Figure 2. After dehydration, only a few particles could be detected in the mixture. This result indicated that most of the ACE-2 NPs were degraded during the process of FD without mannitol present as a cryoprotectant. With the mannitol, a portion of the spherical particles were detected in FD ACE-2 NPs at a mannitol weight ratio of 1:1. SD ACE-2 NPs with mannitol present had weight ratios of 1:1 and 5:1, compared to SFD with same mannitol ratios, showing spherical particles and a smooth surface observed. SD ACE-2 NPs without mannitol retained the spherical structure, but also had irregular, craggy surfaces, while SFD ACE-2 NPs without mannitol present showed a spherical structure with highly porous surfaces.

During FD, mannitol acted as a cryoprotectant and remained in an amorphous form, protecting the ACE-2 NPs from damage caused by ice crystal formation. In contrast, the SD process without a freezing step could promote encapsulation during the dehydration process. Therefore, mannitol was not always required for the SD process, but it could still serve as a bulking agent to give the ACE-2 NPs a more spherical structure. This is seen in SD ACE-2 NPs without mannitol, where particles maintained a spherical structure, but with craggy surfaces (Figure 2). Furthermore, it was revealed that some large particles were detected in all FD, SD, and SFD ACE-2 NP products, containing high amounts of mannitol. This was likely due to the accumulation of mannitol in the particle core together with ACE-2 within the chitosan capsule layer. It can be observed that using SFD also noticeably protected the spherical structure of the ACE-2 NPs. This can be attributed to the quick freezing of the ACE-2 NPs and part of the water from the first spraying step of SFD using liquid nitrogen transferring into fine ice crystals rather than forming undesirable, irregular large ice crystals. Thus, even in the absence of mannitol, the spherical structure and quality of pores on the surface of ACE-2 NPs were acceptable. This was further enhanced with low mannitol (1:1 of mannitol to the total weight of ACE-2 NPs) present and was required for employing the SFD technique in order to obtain ACE-2 NP powders with intact surfaces and smaller particles.

### 3.3. FTIR–ATR Spectroscopy Analysis

**Figure 3:**
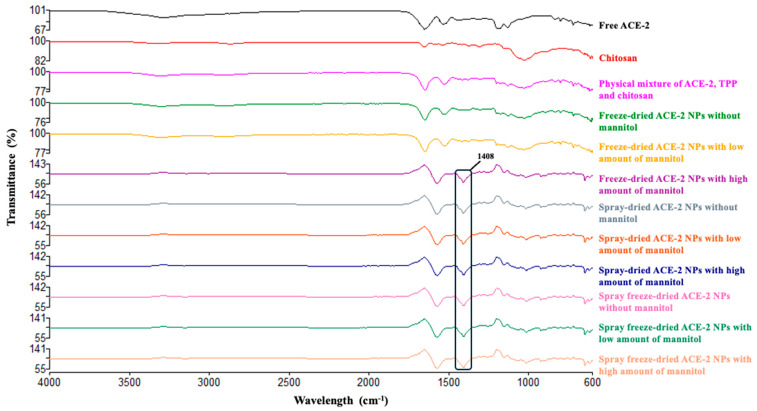
FTIR–ATR spectra of free ACE-2, chitosan, physical mixture of chitosan/TPP/ACE-2, and ACE-2 NPs dehydrated at varying mannitol concentrations by different drying methods. The different conditions used to derive the NPs are provided at the right of the diagram spectrum.

The FTIR–ATR spectroscopy characterized free ACE-2, chitosan, a physical mixture of chitosan, TPP, and ACE-2, and ACE-2-loaded NPs dehydrated using different methods. Band intensities appearing at 1639, 1548, and 1408 cm^−1^ were observed for SD and SFD dehydrated ACE-2 NPs at all mannitol concentrations tested, and also with FD using a mannitol total weight ratio of 5:1 (Figure 3). The observed increase in matrix strength was attributed to the cross-linking between chitosan, TPP, and ACE-2 [19]. The chitosan–ACE-2 interaction shown in the FTIR–ATR spectra reflected the overlapping of the chitosan bands with those of ACE-2, resulting in an increase in the carbonyl intensity band (1639 cm^−1^) and amine band (1548 cm^−1^), respectively. The TPP tripolyphosphate group attached to the ammonium group of chitosan gave rise to a band of 1408 cm^−1^. Furthermore, these results were consistent with those SEM spectra (Figure 2) where ACE-2 NPs remained intact upon SD and SFD at all mannitol concentrations, along with FD samples that had a mannitol total weight of 5:1. In contrast, NPs without mannitol or with the lowest mannitol ratio (e.g., 1:1) showed FTIR–ATR spectra that were very similar to the physical mixture of chitosan, TPP, and ACE-2. From this, we conclude that the cross-linking between chitosan, TPP, and ACE-2 was no longer present in FD ACE-2 NPs in the absence of mannitol or was present at very low ratios. This was confirmed with the SEM findings shown in Figure 2. Since only FD ACE-2 NPs without and with low amounts of mannitol were especially fragile and broken during FD dehydration, we did not include them for subsequent in vitro tests.

### 3.4. Yield Rate, Reconstitution, and Stability Analysis

Dehydrated NPs are typically used for long-term storage or reprocessing into other formulations, and thus, the capacity of dried NPs to reconstitute into water is important for successful formulation. In this study, the results indicated that the yield rate and LC of all SD samples were lower than those treated with FD and SFD (Table 1) (Figure S2). This outcome can be attributed to the adhesion of the powder particles to the drying chamber wall. Since ACE-2 is temperature sensitive, the presence of mannitol and use of a relatively low outlet temperature (40 °C) reduced, to some extent, ACE-2 degradation during the SD process. It was also found that the average particle size of ACE-2 NPs prepared by SD and SFD without the presence of mannitol was preserved after reconstitution. The size of ACE-2 NPs processed by SD and SFD that had small amounts of mannitol (1:1 of its total weight) were only increased slightly after reconstitution. Increasing the amount of mannitol to a maximum ratio (e.g., 5:1) had a significant effect (*p* < 0.05) on the size of ACE-2 NPs prepared by the SD, FD, and SFD dehydration methods, respectively. The use of FD on ACE-2 NPs produced the largest particle sizes upon reconstitution, compared to other drying methods. These results indicate that the use of the bulking agent, mannitol, and control of ice crystal formation before dehydration were important for increasing the particle size of the NPs after reconstitution. In this study, the PDI did not change significantly after reconstitution for any NPs. However, for the EE parameter, after reconstitution, the ACE-2 NPs dehydrated by SFD in the absence of mannitol was largely reduced, thereby signifying that although the spherical structure of the ACE-2 NPs was not changed, the porous surfaces could result in NP leakage of the ACE-2. Furthermore, the addition of mannitol significantly reduced the ACE-2 loading in all NPs tested. It is known that the LC of NPs is an important parameter when assessing potential use for pharmaceutical applications. For NPs with low LC, very large numbers of NPs are required to reach a therapeutic threshold. Considering all parameters assessed in this study, it was clear that the use of SFD to dehydrate ACE-2 NPs with low mannitol (1:1 of its total weight) present was the best set of process conditions used for the reconstitution of ACE-NP. Moreover, these conditions also resulted in a high yield rate, PDI, and EE, respectively, while also maintaining good particle sizes and ACE-2 LC.

### 3.5. In Vitro Release of ACE-2 from NPs at Different pHs

**Figure 4:**
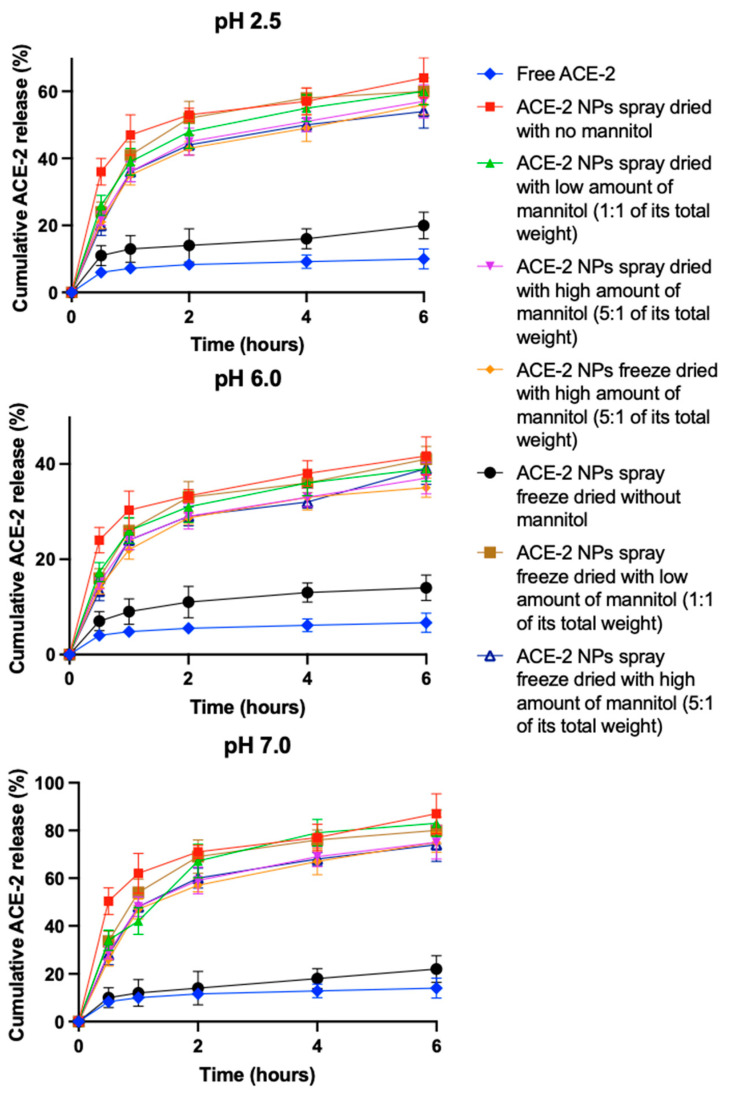
Release behaviors of free ACE-2 and reconstituted ACE-2 NPs dehydrated by different drying methods at different pHs modulating the pH environment of the stomach, duodenum, and upper small intestine. Values represent mean ± SD, *n* = 3.

To simulate the pH environment of the stomach, duodenum, and upper small intestine, respectively, free ACE-2 and redissolved dried ACE-2 NPs were incubated in different buffers (e.g., pH 2.5, 6.0, and 7.0, respectively) with added pepsin (0.5 mg/mL) and incubated at 37 °C in a water bath for 6 h. The results showed first-order kinetics (Table S1). All NPs exhibited an initial release of ACE-2 in the first hour, followed by a slow release over the next 5 h in all buffer solutions that simulated different gastrointestinal environments (Figure 4). This observed rapid initial release was attributed to the high concentration difference at the beginning of the incubation period and the rapid surface desorption of ACE-2 molecules that were not completely anchored to the internal structure of the particles. All NPs exhibited the highest release of ACE-2 at pH 7, followed by pH 2.5 and pH 6.0. This result can be attributed to the fact that the deprotonation of chitosan occurs at higher pHs, thus resulting in a less compact polymer network and greater the release of the loaded ACE-2. Furthermore, based on the kinetic model, the SD-processed ACE-2 NPs (Table S1) without mannitol added showed the fastest release profile, followed by ACE-2 NPs processed by SD and SFD, respectively, both containing the low amount of mannitol (1:1 of its total weight). Release was different between ACE-2 NPs prepared by SD and SFD, each containing a high amount of mannitol (5:1 of its total weight) present, and the FD ACE-2 NPs, which also comprised a high amount of mannitol (5:1 of its total weight). This finding is consistent with the relative order of the particle sizes for redissolved ACE-2 NPs (Table 1), where the smallest particle size had the fastest release rate. Small particles with a larger surface area facilitate the ACE-2 location close to the particle surface, thus resulting in a rapid rate of release. It can also be noted that SFD-processed ACE-2 NPs without mannitol present produced a relatively low ACE-2 release compared with the other treatments (Table S1) (Figure 4), indicating that a highly porous surface and low EE did not protect ACE-2 degradation in the presence of pepsin.

### 3.6. In Vitro Cellular (Cacp-2) Uptake of Dehydrated ACE-2 NPs

**Figure 5:**
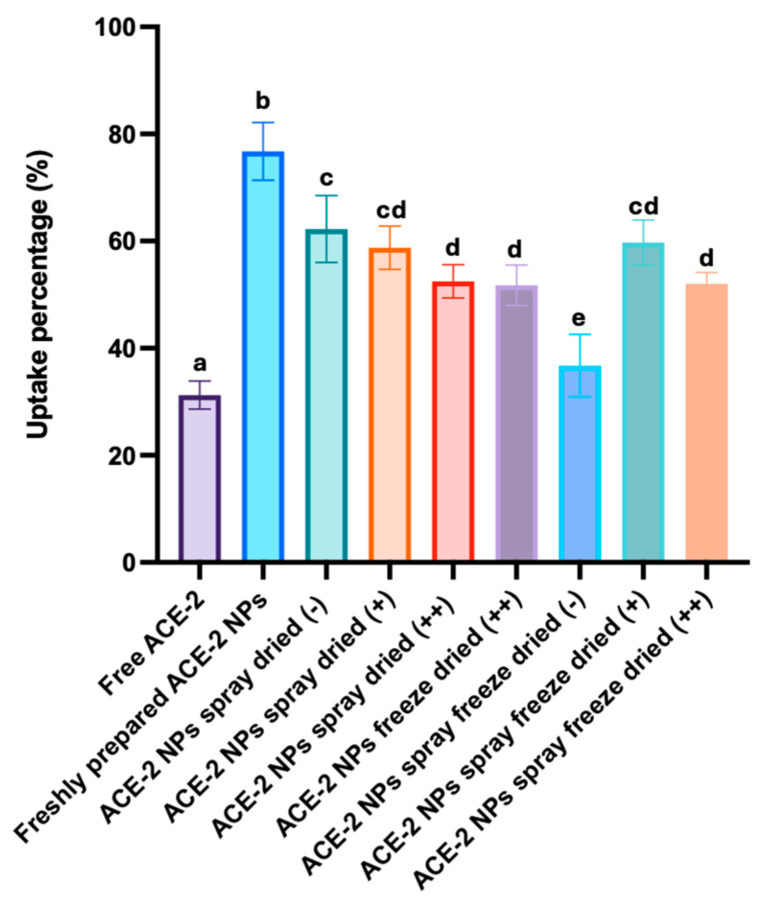
Caco-2 cell relative uptake of ACE-2, freshly prepared ACE-2 NPs, and ACE-2 NPs dehydrated in different ways. − = no mannitol; + = low mannitol; ++ = high mannitol. Values represent mean + SD (*n* = 3). Different superscripts ^a,b,c,d,e^ in rows represent statistical differences (ANOVA; *p* < 0.05).

Free ACE-2 (25 μg/mL), along with an equivalent amount of ACE-2 content, recovered from freshly prepared ACE-2 NPs, and dry-processed ACE-2 NPs were assessed for relative intracellular uptake in Caco-2 cells by cell lysis (Figure 5). The results showed that the SD ACE-2 NPs without mannitol had the highest cellular uptake in differentiated Caco-2 cells, followed by ACE-2 NPs that were dried using SD and SFD, respectively, with low mannitol (1:1 of its total weight). ACE-2 NPs that had high mannitol content (5:1 of its total weight) and SFD-dried product had relatively lower uptake (*p* < 0.05), as was the case with FD ACE-2 NPs that were prepared with high mannitol (5:1 of its total weight). In addition to smaller ACE-2 NPs showing higher cellular uptake, it was also observed that the addition of mannitol at high concentrations lowered cellular uptake, a feature that was likely due to the increased viscosity of the solution that lowers cellular permeability. As the particle surface area increased, the size decreased, and a larger surface area promoted the particle diffusion rate into cells. However, with the increase in the viscosity, the diffusion rate of the nanoparticles decreased, so that the cell uptake of ACE-2 NPs was reduced [20]. It was also observed that although SFD ACE-2 NPs without mannitol had a small particle size and a porous surface, they were restricted in the retention of the ACE-2 NP after being redissolved. Based on the results above, it is noteworthy that despite the fact that SD ACE-2 NPs without mannitol exhibited the highest Caco-2 cell uptake, the SFD ACE-2 with low amounts of mannitol (1:1 of its total weight) was the preferred process for producing ACE-2 NPs because of its relatively high cellular uptake and yield rate.

### 3.7. In Vitro Toxicity Evaluation of Dehydrated ACE-2 NPs

**Figure 6:**
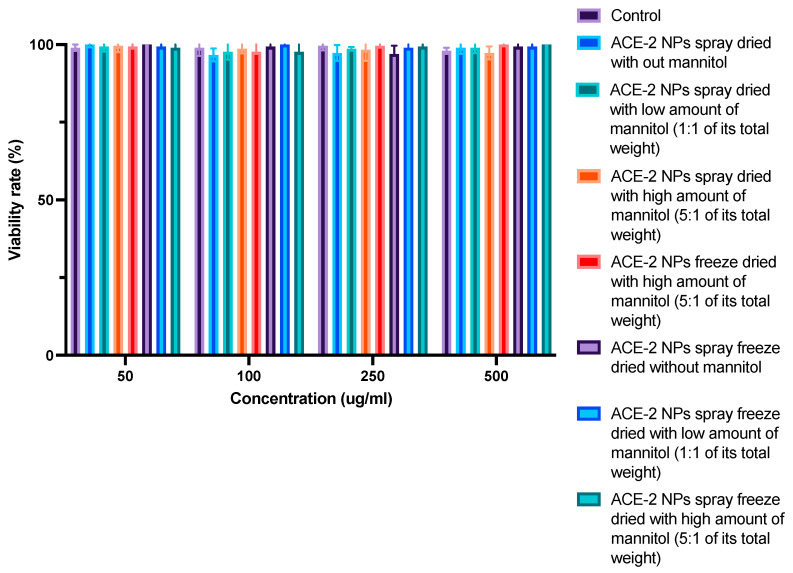
Cell viability (%Control) of differentiated Caco-2 cells treated by ACE-2 NPs dehydrated in different ways. Values represent mean + SD (*n* = 3).

The MTT assay was used to determine the possible cytotoxicity of all dehydrated ACE-2 NPs (Figure 6). Neither free ACE-2 nor dehydrated ACE-2 NPs showed any signs of Caco-2-reduced cell redox balance at concentrations ranging from 50 to 500 μg/mL.

## 4. Conclusions

The present study examined the effects of different processing parameters (e.g., pH, mixing ratio, dehydration method, and mannitol concentration) on the encapsulation efficiency and capacity of synthesized ACE-2 in chitosan/TPP constructed NPs. Employing a pH 5.5 with a chitosan/ACE-2 ratio of 2:1 produced an optimal particle size of 303.7 nm and a 98.4% EE. After dehydration, the spherical structures of ACE-2 NPs remained intact for both SFD- and FD-processed samples with a mannitol weight ratio of 5:1. After reconstitution, however, all SD samples produced a low yield rate, especially the FD ACE-2 NPs that contained high mannitol and the largest particle size. Only the SFD ACE-2 NPs with lows amount of mannitol (1:1 of its total weight) had a high yield rate, similar PDI and EE, and effective particle size for ACE-2 loading. The release behavior of ACE-2 from all dehydrated NPs was rapid at both pH 2.5 and 7 solutions, while greater stability was observed at pH 6.0. The ACE-2 NPs dehydrated using SD without mannitol and SFD using low amounts of mannitol (1:1 of total weight) both had a relatively fast release in media and a high Caco-2 cell uptake. However, considering the relatively low yield rate of using SD dehydration, along with the high cost of ACE-2, it was concluded that the SFD process was most suitable for the production of powdered ACE-2 NPs. The findings of this research will add to the current knowledge available for constructing non-invasive ACE-2 delivery systems utilizing SFD as a dehydration method for optimal applications.

## Figures and Tables

**Table 1:** Physicochemical properties (particles, PDI, entrapment efficiency, loading capacity, zeta potential, and yield rate) of freshly prepared and reconstituted ACE-2 NPs dehydrated by different drying methods (one-way ANOVA comparisons, *p* < 0.05).

	Freshly Prepared	FD ++	SD −	SD +	SD ++	SFD −	SFD +	SFD −
PS (nm)	303 ± 12 ^a^	674 ± 47 ^b^	343 ± 33 ^c^	535 ± 24 ^d^	613 ± 37 ^b^	366 ± 29 ^c^	548 ± 30 ^d^	664 ± 42 ^b^
PDI	0.19 ± 0.02 ^a^	0.24 ± 0.03 ^a^	0.22 ± 0.02 ^a^	0.23 ± 0.03 ^a^	0.25 ± 0.05 ^a^	0.22 ± 0.04 ^a^	0.21 ± 0.03 ^a^	0.20 ± 0.04 ^a^
EE (%)	98.40± 0.32 ^a^	98.01 ± 0.43 ^a^	97.63 ± 0.29 ^a^	99.01 ± 0.51 ^a^	98.23 ± 0.43 ^a^	99.03 ± 0.39 ^a^	98.93 ± 0.36 ^a^	99.02 ± 0.22 ^a^
LC (%)	28.42 ± 0.21 ^a^	4.71 ± 0.13 ^b^	18.14 ± 0.44 ^c^	3.92 ± 0.10 ^d^	2.02 ± 0.06 ^e^	27.84 ± 0.30 ^a^	14.22 ± 0.32 ^f^	4.69 ± 0.36 ^b^
ZP (mV)	6.8 ± 0.2 ^a^	6.5 ± 0.3 ^a^	6.6 ± 0.2 ^a^	7.1 ± 0.3 ^a^	6.5 ± 0.2 ^a^	6.7 ± 0.3 ^a^	6.7 ± 0.4 ^a^	6.8 ± 0.1 ^a^
YR (%)	NA	99.83 ± 0.13 ^a^	47.87 ± 2.13 ^b^	53.32 ± 3.13 ^c^	55.31 ± 2.13 ^c^	99.67 ± 0.13 ^a^	98.98 ± 0.13 ^a^	99.32 ± 0.13 ^a^

Abbreviations: FD, freeze-drying; SD, spray-drying; SFD, spray-freeze-drying; PS, particle size; PDI, polydispersity index; EE, encapsulation efficiency; LC, loading content; YR, yield rate; and NA, not applicable. − = no mannitol; + = low mannitol; ++ = high mannitol. Values represent mean + SD (*n* = 3). Different superscripts ^a,b,c,d,e,f,^ in rows represent statistical differences (ANOVA; *p* < 0.05).

## Data Availability

The original contributions presented in this study are included in the article/Supplementary Material. Further inquiries can be directed to the corresponding author.

## Supplementary Materials

The following supporting information can be downloaded at: https://www.mdpi.com/article/10.3390/pharmaceutics17040537/s1, Figure S1. The particle size distribution (A) and zeta potential (B) of optimized ACE-2 NPs. Figure S2. Particle size distribution of fresh prepared ACE-2 NPs (A), redissolved ACE-2 NPs dehydrated by FD ++ (B), SD − (C), SD + (D), SD ++ (E), SFD − (F), SFD + (G) and SFD ++ (H). Table S1. First-order regression parameters of release behavior fixed by (Released ACE-2(t) = a*ln(t) + b), where a represent the release speed, while b represents the release rate.
