# Supplementary material for: Comparison of Drying Techniques to Produce Stable and Bioavailable Encapsulated ACE-2 Nanoparticles
Source: Pharmaceutics. 2025 Apr 21;17(4):537. doi: 10.3390/pharmaceutics17040537 (PMC12030647; doi:10.3390/pharmaceutics17040537)
Supplement: Supplementary file 1 [file pharmaceutics-17-00537-s001.zip › pharmaceutics-3565486-supplementary.pdf]

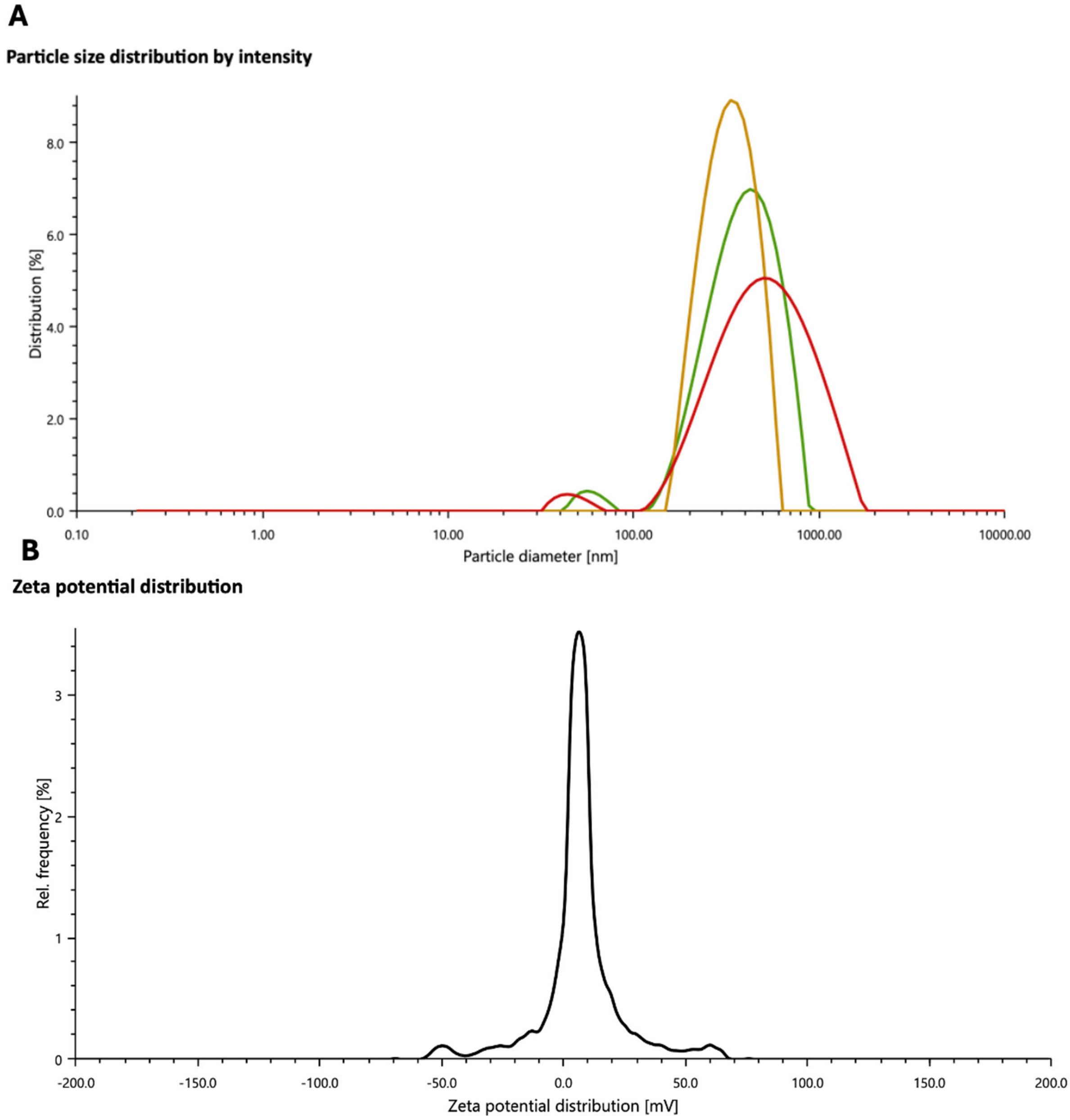

Figure S1. The particle size distribution (A) and zeta potential (B) of optimized ACE-2 NPs

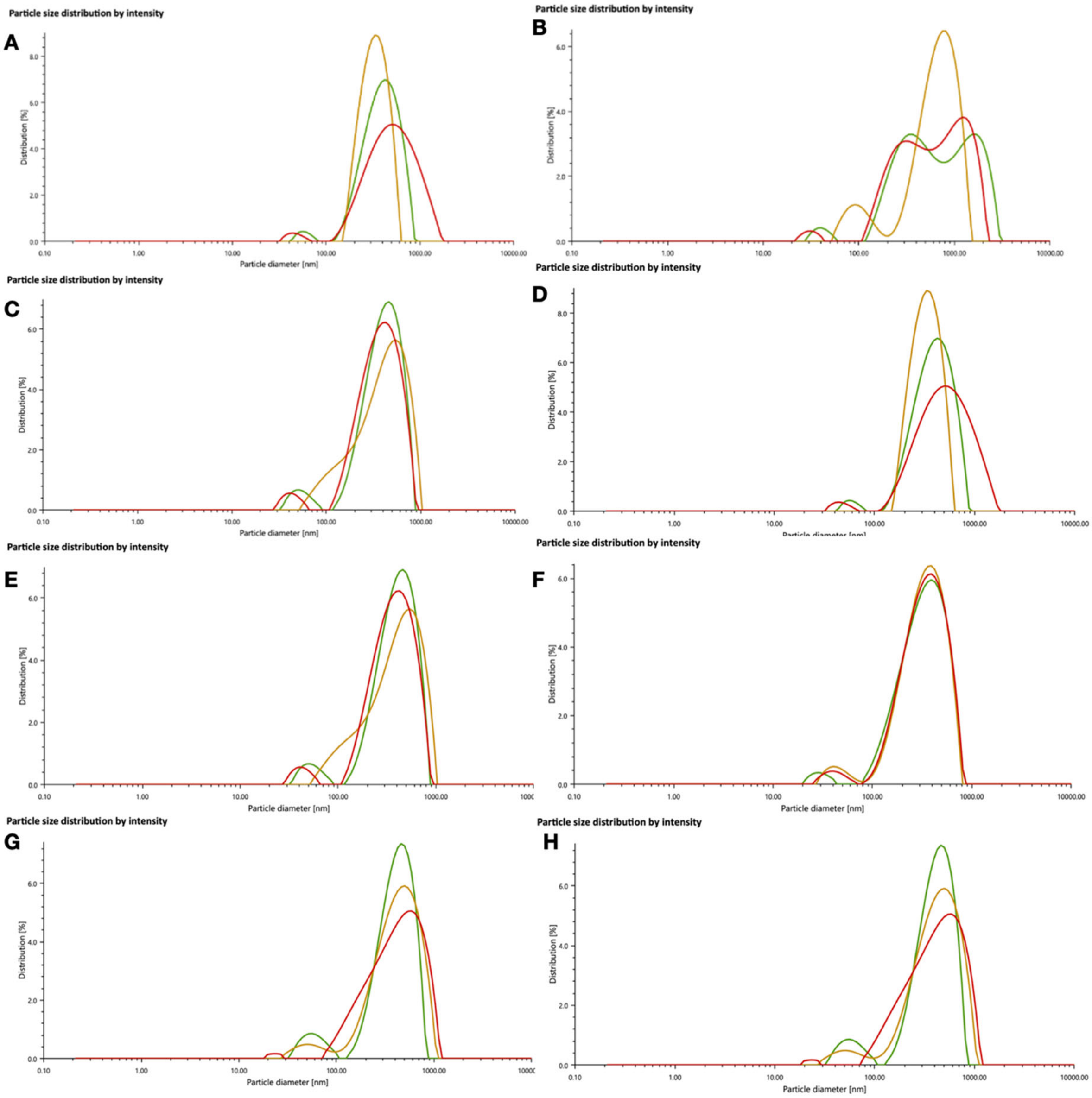

Figure S2. Particle size distribution of fresh prepared ACE-2 NPs (A), redissolved ACE-2 NPs dehydrated by FD ++ (B), SD – (C), SD + (D), SD ++ (E), SFD – (F), SFD + (G) and SFD ++ (H)

Table S1. First-order regression parameters of release behavior fixed by (Released ACE-2(t)= a*ln(t)+b), where a represent the release speed, while b represents the release rate.

	a	b	R^2
PH=2.5			
Free ACE-2	1.57	7.14	0.9973
SD –	14.23	44.83	0.9698
SD +	13.28	37.74	0.9848
SD ++	13.42	33.67	0.9770
FD ++	13.22	32.40	0.9769
SFD –	3.21	12.86	0.9848
SFD +	13.44	37.96	0.9369
SFD ++	13.02	32.93	0.9530
PH=6.0			
Free ACE-2	1.32	6.43	0.9874
SD –	10.02	28.32	0.9562
SD +	9.45	23.42	0.9483
SD ++	9.28	21.57	0.9576
FD ++	9.33	23.42	0.9666
SFD –	2.87	11.53	0.9563
SFD +	9.50	24.84	0.9753
SFD ++	9.32	22.43	0.9812
PH=7.0			
Free ACE-2	1.58	7.87	0.9674
SD –	19.02	49.37	0.9573
SD +	17.92	43.66	0.9345
SD ++	17.89	44.63	0.9835
FD ++	17.76	42.56	0.9257
SFD –	4.03	13.56	0.9958
SFD +	18.04	45.80	0.9583
SFD ++	17.91	42.21	0.9574
